# Uridine and pyruvate supplementation is associated with Th1 polarization during mitotoxic antibiotic therapy

**DOI:** 10.1016/j.isci.2026.117230

**Published:** 2026-08-24

**Authors:** Stefania De Santis, Monica Rutigliano, Federica Simone, Letizia Larocca, Maria Laura Matrella, Martina Milella, Antonio Vavallo, Pasquale Ditonno, Nicola Marrano, Anna Mestice, Stefano Battaglia, Michele Battaglia, Antonio Moschetta, Peter Seibel, Elena Piccinin, Gaetano Villani

**Affiliations:** 1 Department of Translational Biomedicine and Neuroscience, University of Bari Aldo Moro, Bari, Italy; 2 Department of Precision and Regenerative Medicine and Ionian Area, University of Bari Aldo Moro, Bari, Italy; 3 Department of Interdisciplinary Medicine, University of Bari Aldo Moro, Bari, Italy; 4 Molecular Cell Therapy, BBZ, Medical Faculty, University of Leipzig, Leipzig, Germany

**Keywords:** immunometabolism, OXPHOS, antibiotics, T cells, mitochondria, GDF15, uridine, pyruvate

## Abstract

Antibiotics with off-target mitochondrial toxicity can impair host immunity, yet their impact on human adaptive immunity *in vivo* remains unclear. Because T cell activation and differentiation depend on mitochondrial metabolism, antibiotic-induced mitochondrial stress may alter T helper cell function. We investigated whether uridine and pyruvate (UP) supplementation modulates immune responses in patients receiving mitotoxic antibiotics. In a pilot observational study, 67 patients undergoing prophylactic antibiotic therapy received either antibiotics alone or antibiotics plus daily UP supplementation. Antibiotic exposure increased circulating growth differentiation factor-15 (GDF15), indicating mitochondrial stress, and altered T cell response. UP supplementation was associated with immune features consistent with preserved inflammatory competence, including a trend toward Th1 polarization. These findings suggest that mitotoxic antibiotics can influence human T cell programs and support the hypothesis that UP supplementation may preserve pro-inflammatory T cell responses during antibiotic therapy, representing a potential metabolic strategy to mitigate antibiotic-induced immunotoxicity.

## Highlights

• Mitotoxic antibiotics increase circulating growth differentiation factor-15
• Metabolic support may preserve immune competence during antibiotic therapy
• Uridine and pyruvate supplementation promote Th1-skewed immune responses

## Graphical abstract

**Figure:**
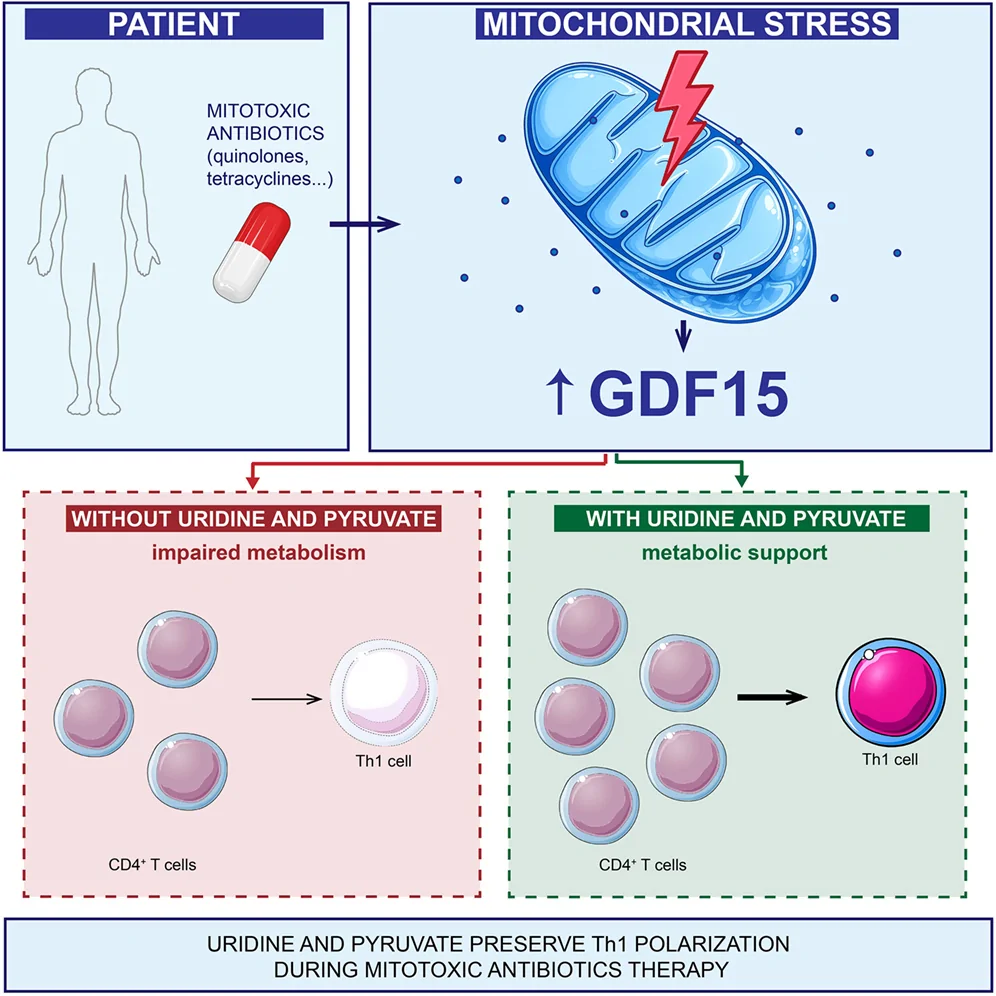

## Teaser

Biological sciences; Human metabolism; Immunology; Immune system; Immune response

## Introduction

Antibiotics are the primary treatment against bacterial infections. However, the global emergence of antimicrobial resistance^1^ represents a critical public health threat. Addressing this challenge requires a comprehensive understanding of antibiotic-induced effects beyond their direct bactericidal activity, particularly in relation to host immunity. Indeed, it has been suggested that the interaction between the strength of the immune response and the pathogen load can affect the overall bactericidal effect, even when different antibiotic doses are used.^2^

Notably, certain antibiotics can modulate the functions of immune cells and inhibit pro-inflammatory molecules production.^3^^,^^4^ A growing body of evidence attributes these immunomodulatory effects to “mitotoxicity,” defined as the off-target impact of certain antibiotics on host cell mitochondria.^5^^,^^6^^,^^7^ Indeed, due to the endosymbiotic origin of mitochondria, some antibiotics can impair mitochondrial nucleic acid, protein synthesis and/or transport pathways, compromising host cell functionality.^8^^,^^9^^,^^10^ This is highly relevant for immune cells, which rely on mitochondrial function for energy production, signaling, and pathogen clearance. Consequently, antibiotic-induced mitochondrial dysfunctions can compromise the efficacy of immune cells, potentially hindering infection resolution and causing well-known adverse effects of antibiotics, such as neutropenia and hypersensitivity.^3^^,^^11^^,^^12^^,^^13^ Although antibiotic-induced mitochondrial dysfunction has been demonstrated *in vitro* and in animal models, its impact on human immune cell function *in vivo* remains poorly understood. Understanding how mitotoxic antibiotics influence immune cell programs is particularly relevant because immune competence plays a critical role in infection clearance and may influence treatment outcomes.

T cells, central to adaptive immunity, rely on distinct mitochondrial metabolic programs to sustain their differentiation and function. Effector populations with high oxidative demands, including Th1, Th17, and cytotoxic T cells, depend on intact oxidative phosphorylation to sustain interferon γ (IFN-γ) production, cytotoxic activity, and long-term persistence.^14^ In contrast, Th2 cells rely more heavily on glycolytic pathways and are comparatively less sensitive to mitochondrial impairment, which may result in their preferential survival and expansion under conditions of metabolic stress.^15^ Mitochondrial metabolism underpins these distinct CD4^+^ programs by supplying the bioenergetic and biosynthetic requirements for activation, clonal expansion, and effector differentiation.^16^ Mitotoxic antibiotic-induced dysregulations can thus create a selective vulnerability, reshaping the balance among CD4^+^ subsets, skewing the Th1/Th2 ratio, and potentially attenuating cytotoxic immunity and IFN-γ-dependent host defense. Understanding how mitochondrial stress shapes T cell fate is therefore essential.

Previous work from our group showed that supplementation with uridine and pyruvate (UP) can restore the proliferative capacity of T cells exposed to mitotoxic antibiotics.^17^ While uridine overrides the dihydroorotate dehydrogenase stalling, pyruvate bypasses mitochondrial dysfunction and supplies the cellular energetic requirement, leading to an increase in aerobic glycolysis.^18^

In the present study, we investigated whether supplementation with UP during antibiotic therapy modulates immune responses in humans. Specifically, we examined whether metabolic support influences inflammatory cytokine production and CD4^+^ T cell polarization. In addition, we assessed circulating levels of growth differentiation factor-15 (GDF15), a stress-induced mitokine that has emerged as a robust biomarker of mitochondrial dysfunction.^19^^,^^20^ Overall, we provide evidence that mitotoxic antibiotics induce systemic mitochondrial stress and that metabolic supplementation with UP promotes a shift toward Th1 immune responses.

## Results

### Mitotoxic antibiotics increase GDF15 levels in patients’ sera

**Figure 1:**
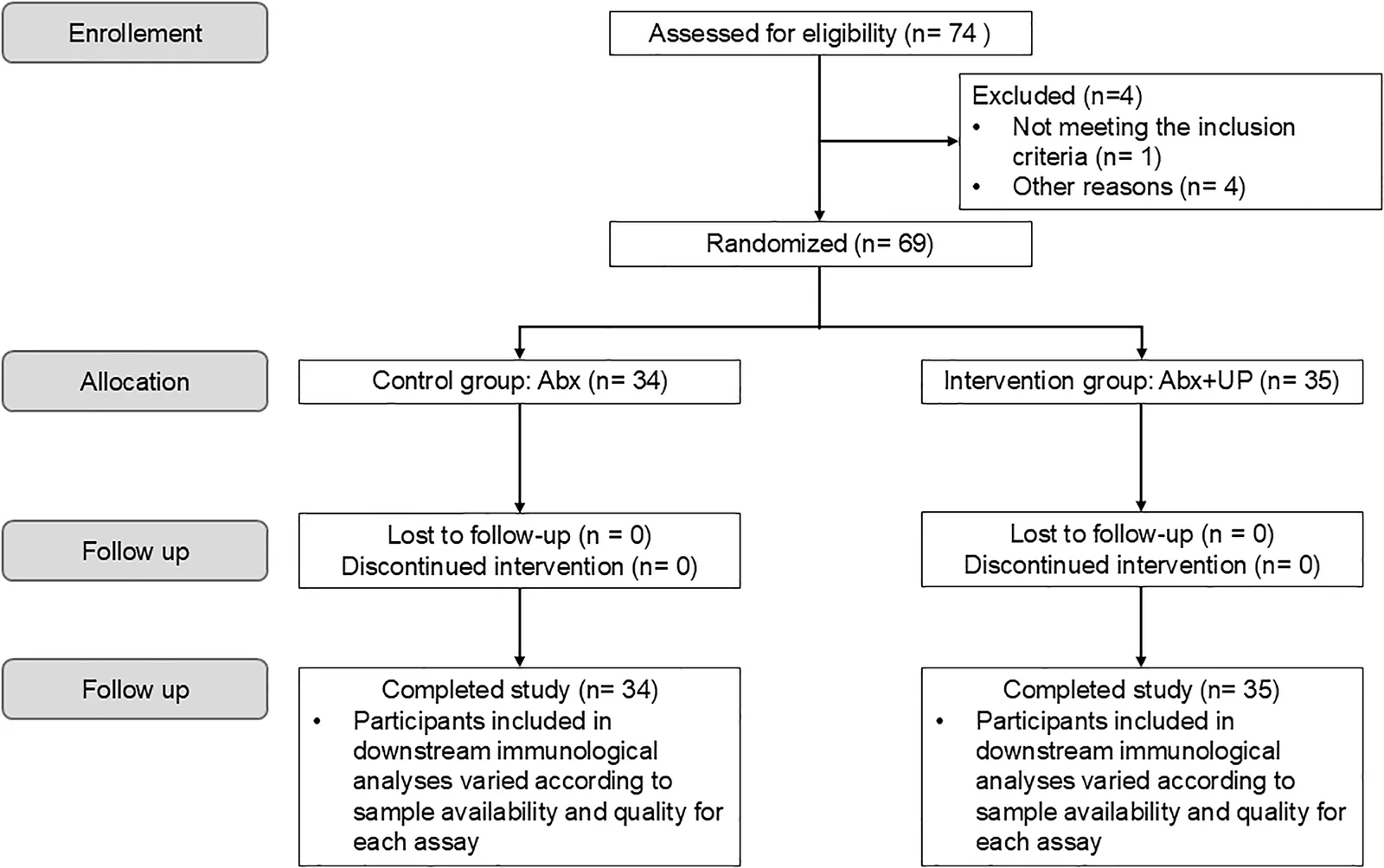
Flow chart for the selection of study population

Asymptomatic bacteriuria and/or positive sperm culture are common situations in clinical practice, often not associated with overt symptoms, including inflammation. Although the antibiotic treatment is not indicated for these conditions, the patients we enrolled in the study underwent specific therapy for the prophylaxis of invasive urological procedures (Figure 1 and Table 1).

**Table 1:** Demographic and clinical characterization of the study population

Variables	Abx	Abx + UP
Number	34	35
Age	52.1 ± 16.9	56.4 ± 16.4
BMI	25.6 ± 3.2	25.8 ± 3.1
WBC (10^3^/μL)	5.85 ± 1.31	6.58 ± 1.30
Neutrophiles (10^3^/μL)	2.74 ± 1.24	3.04 ± 1.47
Monocytes (10^3^/μL)	0.44 ± 0.20	0.46 ± 0.15
Lymphocytes (10^3^/μL)	2.05 ± 0.74	2.42 ± 0.61

**Sex**		

Female	7 (20.6)	9 (25.7)
Male	27 (79.4)	26 (74.3)

**Smoke**		

Smokers	6 (18.2)	4 (11.4)
Non-smokers	27 (81.8)	31 (88.6)

**General comorbidity**		

Presence	14 (41.2)	15 (44.1)
Absence	20 (58.8)	19 (55.9)

**Nephrological disease**		

Presence	3 (8.8)	2 (5.9)
Absence	31 (91.2)	32 (94.1)

**Nephrolithiasis**		

Presence	15 (44.1)	15 (44.1)
Absence	19 (55.9)	19 (55.9)

**Antibiotic treatment**		

Quinolones	24	23
Macrolides	2	0
Oxazolidinones	1	1
Tetracyclines	7	9

Numeric variables are expressed as mean ± standard deviation. Categorical variables are expressed as mean (%). Each item was compared among the two groups using Welch’s or Mann-Whitney’s *U* test for quantitative variables and Pearson χ^2^ test for categorical ones. For the variable smoke, the data of one patient was not available. Abx, patients receiving antibiotic treatment alone; Abx + UP, patients receiving antibiotic therapy plus UP supplementation; BMI, body mass index; WBC, white blood cells count at the end of treatment; Neutrophiles, neutrophiles count at the end of treatment; monocytes, monocytes count at the end of treatment; lymphocytes, lymphocytes count at the end of treatment.

**Figure 2:**
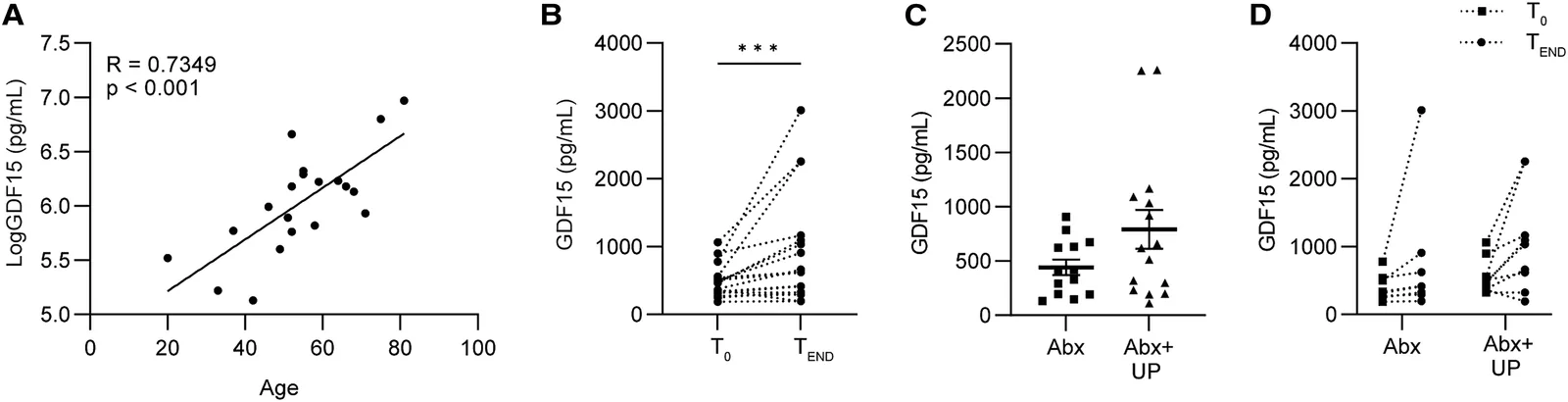
Mitotoxic antibiotics increased GDF15 levels in patients’ sera Growth differentiation factor-15 (GDF15) levels were evaluated in patients’ sera before (T0) or after (TEND) mitotoxic antibiotic (Abx) administration, with or without uridine and pyruvate (UP) supplementation. (A) Correlation of GDF15 at T_0_ and patients’ age. (B) Level of GDF15 at T_0_ and T_END_ for patients treated with Abx and Abx + UP. (C) GDF15 level at T_END_ comparing Abx and Abx + UP patients. (D) Level of GDF15 at T_0_ and T_END_ for patients treated with Abx or Abx + UP. Data are shown as mean ± SEM (*n* = 15/group). Comparison between groups was performed by one-way Anova, followed by the Kruskal-Wallis test for multiple comparisons (∗∗∗*p* < 0.001). T_0_, before antibiotic treatment; T_END_, last day of antibiotic treatment. Abx, patients receiving antibiotic treatment alone; Abx + UP, patients receiving antibiotic therapy plus UP supplementation.

To determine whether antibiotic therapy induces mitochondrial stress *in vivo*, we quantified circulating levels of GDF15, a mitokine widely recognized as a biomarker of mitochondrial dysfunction.^19^^,^^21^ The secretion of mitokines, such as GDF15, during cellular stress may indeed reflect the extent of the mitochondrial damage. Consistent with previous reports linking GDF15 levels with aging,^22^ baseline GDF15 concentrations positively correlated with patient age (Figure 2A). Importantly, serum GDF15 levels increased significantly following 7–10 days of antibiotic therapy, indicating activation of a mitochondrial stress response during treatment in both Abx- and Abx + UP-treated patients (Figure 2B). As expected, this increase was observed irrespective of UP supplementation (Figures 2C and 2D), thus suggesting that UP supplementation does not directly restore mitochondrial function but rather provides a metabolic bypass that supports cellular processes under mitochondrial stress. Collectively, the increase in circulating GDF15 following antibiotic exposure suggests activation of a mitochondrial stress response *in vivo*.

### UP supplementation rescues the production of proinflammatory molecules

**Figure 3:**
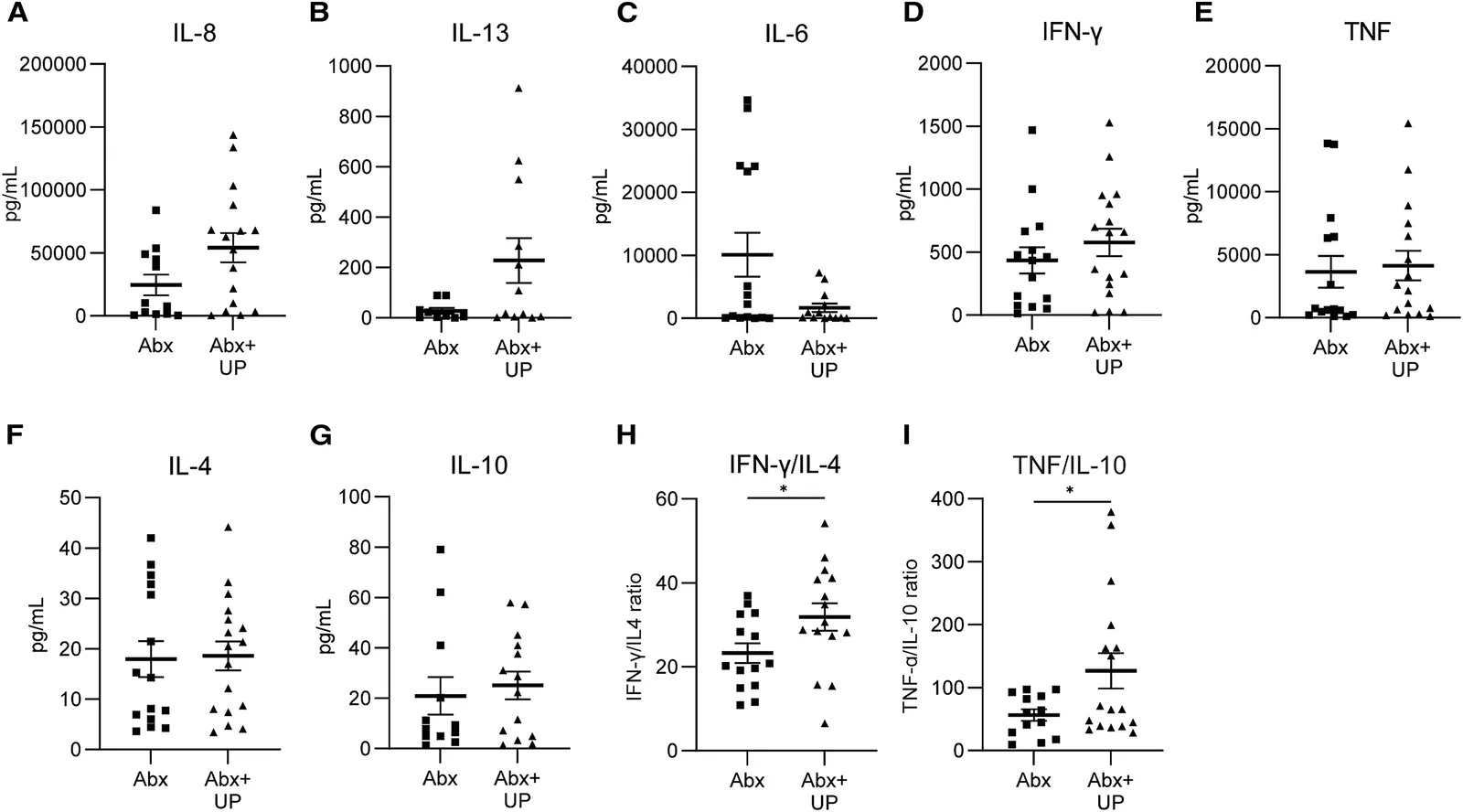
UP supplementation during mitotoxic antibiotics modulates the release of proinflammatory molecules (A–G) Cytokine and growth factor concentrations in culture supernatants from PBMCs isolated at the end of antibiotic treatment from patients treated with Abx alone vs. Abx + UP. PBMCs were stimulated to differentiate into T cells, cultured in autologous serum for 96 h, and cytokine production was quantified by BioPlex analysis. Comparisons between groups were performed using Welch’s *t* test or the Mann-Whitney *U* test after assessment of data normality. To account for multiple testing across the cytokine panel, *p* values were adjusted using the two-stage Benjamini, Krieger, and Yekutieli false discovery rate (FDR) procedure (Q = 5%). No cytokine remained statistically significant after FDR correction. (H and I) Proinflammatory/anti-inflammatory cytokine ratio in patients treated with Abx alone vs. Abx + UP. Data are shown as mean ± SEM (*n* = 15–17/group). Comparisons between groups were performed using Welch’s *t* test or the Mann-Whitney *U* test after assessment of data normality (∗*p* < 0.05). Abx, patients receiving antibiotic treatment alone; Abx + UP, patients receiving antibiotic therapy plus UP supplementation.

In our previous study,^17^ we demonstrated that administration of UP to patients treated with mitotoxic antibiotics could sustain and protect the proliferative capacity of CD3^+^ T cells. However, whether UP supplementation can elicit a specific subset of T helper cells was not addressed. Nowadays, it is well recognized that Th1 cells, under the control of TBX21/T-bet, tend to produce pro-inflammatory cytokines (i.e., IFN-γ) and mediate protective immunity against intracellular pathogens,^23^^,^^24^ while Th2 cells, driven by GATA3, produce interleukin 4 (IL-4), IL-5, and IL-13 and play central roles in defense against extracellular parasites but also contributing to allergic inflammation and asthma.^25^^,^^26^ Previous studies suggest that pyruvate supplementation can sustain glycolytic ATP production when mitochondrial respiration is compromised. Thus, we evaluated whether UP supplementation in patients treated with mitotoxic antibiotics rescues proinflammatory or anti-inflammatory cytokine release. To this end, peripheral blood mononuclear cells (PBMCs) isolated from Abx + UP and Abx groups at the end of the antibiotic treatment (T_END_) were cultured in autologous serum and stimulated to differentiate into T cells. The cell supernatant was then used to perform a Bioplex assay to screen multiple cytokine, chemokine, and growth factor concentrations simultaneously. Evaluation of inflammatory mediators in the primary dataset revealed a selective effect of UP supplementation during antibiotic treatment. Specifically, IL-8 and IL-13 showed higher levels in the Abx + UP group compared with Abx alone, while IL-6 showed a decrease (Figures 3A–3C), indicating a differential modulation across cytokines rather than a uniform directional change. Although the trend toward reduced IL-6 appeared to diverge from the overall pattern of restored inflammatory competence, it likely reflects selective modulation of individual inflammatory mediators rather than a generalized effect on cytokine production. However, none of these differences remained statistically significant after false discovery rate (FDR) correction for multiple comparisons, and these findings should therefore be interpreted as exploratory. Consistent with this, analysis of individual cytokines showed no significant differences between Abx and Abx + UP groups for canonical Th1 (IFN-γ, TNF), Th2 (IL-4), or regulatory (IL-10) mediators (Figures 3D–3G), and no additional changes were observed across other cytokines (Table S2). However, because immune responses depend on the balance between pro- and anti-inflammatory signals rather than absolute cytokine levels alone, we next examined cytokine ratios.^27^^,^^28^^,^^29^ The ratio between anti- and pro-inflammatory cytokines allows the correction for possible interindividual biases. In this context, both IFN-γ/IL-4 and TNF/IL-10 ratios were significantly increased in the Abx + UP group (Figures 3H and 3I), consistent with a relative shift toward a Th1-skewed profile.

Subsequently, we aimed to determine whether a distinct inflammatory response exists based on the antibiotic used, distinguishing between those affecting mtDNA transcription and replication (Abx1) and those impairing the mitochondrial translation process (Abx2). Intriguingly, cytokine, chemokine, and growth factor levels were consistently decreased in patients treated with Abx2 compared with Abx1 (Figure S1), with a significantly lower expression in Abx2 than Abx1 for macrophage inflammatory protein 1α (MIP-1α), IL-15, fibroblast growth factor (FGF), and vascular endothelial growth factor (VEGF) (Figures S1A–S1D), thus suggesting that mitochondrial translation inhibition attenuates the release of newly synthesized cytokines. Intriguingly, UP supplementation was able to rescue the inflammatory response inhibited by Abx2, especially significantly inducing the production of pro-inflammatory cytokines (IL-15, IL-8, IL-4, and IL-13) (Figures S1B, S1F, S1I, and S1L), chemokines (MIP-1α and GM-CSF) (Figures S1A and S1G), and growth factors (FGF and VEGF) (Figures S1C and S1D). Of note, considering the UP supplementation, the expression of GM-CSF and IP-10 is higher in Abx2 than in Abx1 (Figures S1G and S1M). On the contrary, UP supplementation did not significantly affect cytokine release in Abx1-treated specimens, except for IL-6, whose levels were significantly reduced following UP supplementation (Figure S2K). This selective response further supports a differential modulation of inflammatory mediators rather than a uniform effect of UP on cytokine production.

Overall, these data suggest that UP supplementation enhances the production of pro-inflammatory cytokines, especially when an antibiotic impairing the mitochondrial translation process was used.

### UP supplementation sustains Th1 cells’ polarization

**Figure 4:**
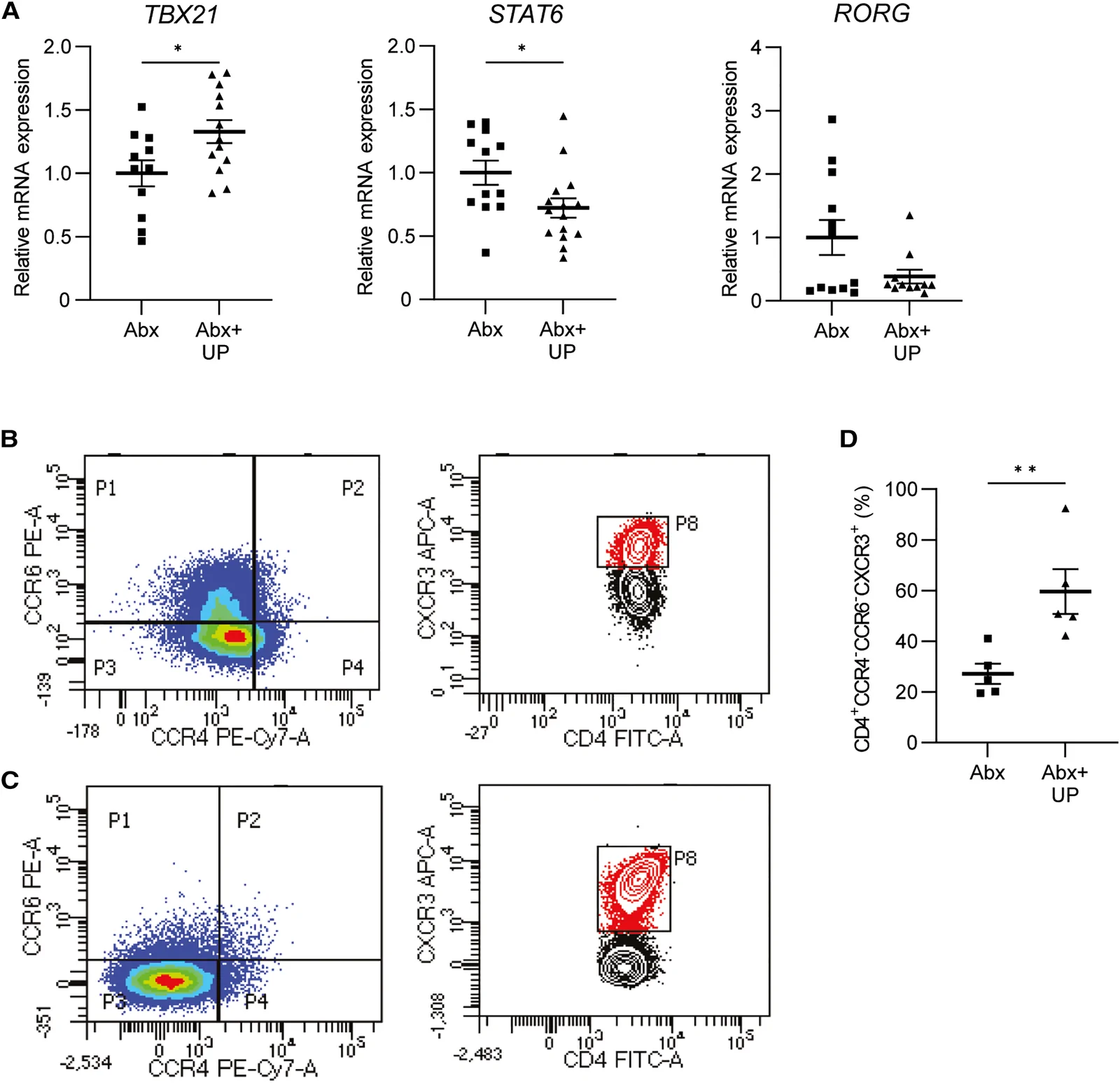
UP supplementation during mitotoxic antibiotics sustains Th1 cells’ polarization (A) Real-time qPCR analysis of PBMCs collected from Abx and Abx + UP patients at the end of antibiotic treatment for transcription factor involved in Th polarization, TBX21 (Th1), STAT6 (Th2), and RORC (Th17). PBMCs were isolated at the end of antibiotic treatment from patients treated with Abx alone vs. Abx + UP, stimulated to differentiate into T cells, and cultured in autologous serum for 96 h. (B–D) Representative contour plots from PBMCs of patients treated with Abx alone (B) or Abx + UP (C). After exclusion of CCR4^+^ (Th2) and CCR6^+^ (Th17) subsets, Th1 cells were identified within the CD4^+^CCR6^−^CCR4^−^ population based on CXCR3 expression. (D) Quantification of CD4^+^CCR6^−^CCR4^−^CXCR3^+^ Th1 cells in Abx vs. Abx + UP. Data are shown as mean ± SEM (*n* = 5–17/group). Comparison between groups was performed by Welch’s test or Mann-Whitney *U* test after assessing the normality (∗*p* < 0.05 and ∗∗*p* < 0.01). Abx, patients receiving mitotoxic antibiotic treatment alone; Abx + UP, patients receiving mitotoxic antibiotic therapy plus UP supplementation.

To determine whether the cytokine changes reflected altered T helper cell differentiation, we analyzed the expression of transcription factors associated with distinct CD4^+^ T cell lineages in PBMCs isolated from Abx + UP and Abx groups at the end of antibiotic treatment (T_END_). In patients treated with Abx + UP, the UP supplementation significantly increased the expression of TBX21 (encoding T-bet), while the expression of STAT6 was reduced (Figure 4A). No significant changes were observed in RORC (encoding RORγt), the master regulator of Th17 cells (Figure 4A). The enrichment of TBX21 expression supports the cytokine data and suggests that UP supplementation promotes activation of Th1-associated transcriptional programs.

To demonstrate that the transcriptional profile contributes to T cells toward Th1 polarization, we analyzed CD4^+^ T cell subsets by flow cytometry in a subset of patients (*n* = 5 per group). Specifically, PBMCs were cultured in autologous serum and stimulated to differentiate into T cells. To selectively identify Th1 cells, CCR6^+^ cells (corresponding to Th17 and related subsets) and CCR4^+^ cells (enriched for Th2 cells) were excluded, thereby focusing the analysis on CD4^+^CCR4^−^CCR6^−^ cells. Within this population, we then assessed CXCR3 expression, a chemokine receptor selectively associated with Th1 polarization and effector function. Patients receiving UP supplementation exhibited a higher frequency of Th1 cells (CD4^+^CCR4^−^CCR6^−^CXCR3^+^), as shown by representative contour plots from Abx (Figure 4B) and Abx + UP (Figure 4C) group. Quantification showed a higher enrichment of Th1 cells in the UP group (59.60 ± 8.84%) compared with antibiotics alone (27.20% ± 3.99%; *p* < 0.01) (Figure 4D). Despite the limited sample size and the need for confirmation in a larger cohort, these results are consistent with a shift toward a Th1-skewed immune profile with UP supplementation during antibiotic therapy.

## Discussion

Antibiotics with off-target effects on mitochondria pose a risk to the immune system, particularly T lymphocytes, which highly rely on mitochondrial fitness to support their activation, clonal expansion, and effector differentiation.^3^^,^^16^^,^^30^ Disruption of T cell mitochondrial function by antibiotics is known to compromise Th1 and CD8^+^ effector responses,^7^^,^^31^ potentially weakening IFN-γ-driven host defense and altering the balance between pro- and anti-inflammatory responses,^32^ with broader implications for antimicrobial resistance.

Here, we performed a pilot study on patients affected by asymptomatic bacteriuria or positive sperm culture, which, despite not being associated with inflammation or other overt symptoms, require antibiotic therapy. We identified the mitokine GDF15, usually secreted upon mitochondrial damage, as a promising new biomarker of antibiotic-induced mitochondrial dysfunction. As expected, the level of GDF15 is increased in older patients, highlighting a possible decline in mitochondrial function with aging. Our data show that circulating GDF15 levels increased after 7–10 days of treatment with a mitotoxic antibiotic, supporting its use for monitoring this side effect, similar to its established role in mitochondrial disorders and aging.^19^^,^^21^^,^^33^ Although an increase of GDF15 has been observed during inflammation and sepsis,^34^ our patients cohort was characterized by conditions not associated with overt symptoms, including inflammation. Given the variability observed in GDF15 levels, larger studies will be required to strengthen this correlation. Moreover, it would be intriguing to understand whether prolonged mitotoxic antibiotic exposure leads to a higher serum GDF15 and if the GDF15 increase observed during infective/inflammatory conditions correlates with the use of these antibiotics in selected patient cohorts.^35^^,^^36^

Our prior work established that UP supplementation counteracts the negative effect of mitotoxic antibiotics, specifically by rescuing T cell proliferation.^17^ Our new findings show that UP supplementation in this context may contribute to restoring pro-inflammatory molecule levels and supporting T cell proliferative responses. One possible explanation is that pyruvate supplementation supports metabolic pathways that partially compensate for mitochondrial dysfunction (i.e., aerobic glycolysis), thereby maintaining T cell activation. This is consistent with the metabolic profile of T-helper subsets: Th2 cells are highly dependent on OXPHOS, therefore being more sensitive to mitotoxic drugs, whereas Th1 cells have a greater reliance on aerobic glycolysis via lactate dehydrogenase (LDH).^15^ Intriguingly, LDHA is induced during T cell activation to support glycolysis and maintain high levels of acetyl-CoA to enhance histone acetylation and transcription of IFN-γ.^37^ It is therefore possible that pyruvate supplementation allows Th1 cells polarization by sustaining glycolysis through mitochondrial-independent oxidation of NADH.

T helper cell commitment to a specific effector state is essential at the onset of an immune reaction, significantly affecting the type and strength of the response, with direct consequences for disease outcome.^38^ Th1 cells drive the clearance of intracellular pathogens. Thus, by supporting the polarization toward Th1 cells, UP supplementation contributes to the eradication of the bacteria by sustaining the function of the immune system. Of note, the use of cytokine ratios (IFN-γ/IL-4 and TNF/IL-10) better captures the functional balance between opposing immune pathways, which may shift even when individual cytokine levels change only modestly, allowing the correction for interindividual variance. By integrating both signals, the ratio can be more sensitive to coordinated biological changes than either marker alone.^39^ Thus, the ratio does not reflect a strong isolated induction of IFN-γ or TNF, but rather a relative change in the balance between Th1- and Th2-associated cytokine signals. The nominal increase in IL-13 observed with UP supplementation may appear unexpected in the context of a relative Th1-skewed profile. However, IL-13 can also be induced as part of tissue-protective or regulatory responses during inflammation and may reflect a compensatory mechanism aimed at limiting excessive inflammatory activation. The overall cytokine pattern suggests the coexistence of pro-inflammatory and compensatory regulatory signals during metabolic rescue rather than a coordinated polarization toward a single T helper phenotype.

We observed that antibiotics inhibiting mitochondrial translation have a more profound effect on inflammatory cytokine expression than those affecting DNA synthesis and transcription processes, suggesting high sensitivity of immune programs to mitochondrial protein synthesis defects. In this context, UP supplementation was associated with nominally higher IL-8 and reduced IL-6 levels. Although the trend toward lower IL-6 appeared to diverge from the overall restoration of inflammatory competence observed throughout the study, this response was isolated and should be interpreted in the context of the broader immunological profile, which included increased Th1 polarization, higher pro-/anti-inflammatory cytokine ratios, and restoration of multiple inflammatory mediators following mitochondrial translation inhibition. A tentative explanation for their divergence could be a differential sensitivity to metabolic cues. In fact, IL-8 production is closely linked to glycolytic flux and NF-κB-dependent pathways, which may be supported by pyruvate-mediated maintenance of cytosolic redox balance under conditions of impaired mitochondrial respiration.^40^ In contrast, IL-6 is strongly influenced by STAT3-dependent feedback loops, which have been reported to involve mitochondrial signaling components.^41^^,^^42^^,^^43^ It is possible that mitochondrial perturbation, combined with metabolic supplementation, differentially affects STAT3 activity and downstream IL-6 amplification, while preserving or enhancing pathways leading to IL-8 production. Importantly, this proposed mechanism remains speculative, and further studies will be necessary to validate this hypothesis and define the molecular pathways responsible for the differential regulation of IL-8 and IL-6 following UP supplementation. Therefore, the divergent IL-6 response should be interpreted as evidence of selective regulation of inflammatory signaling pathways rather than as an indication of globally impaired inflammatory competence.

Thus, our results provide evidence that mitotoxic antibiotics can alter immune programs in humans and identify metabolic supplementation with UP as a strategy capable of restoring pro-inflammatory T cell responses.

While antibiotics are critical for the eradication of pathogen infection, their misuse and the high mutational rate of bacteria drive antimicrobial resistance. This is aggravated by specific antibiotics that can damage mitochondria in immune cells and thereby compromise the host immune system’s ability to fight against pathogens. Our findings show that the adjuvant supplementation of UP is a valid ally of the immune system, as it supports cell metabolism and promotes the proliferation of CD4^+^ Th1 cells, thus helping pathogen eradication. Future studies should test whether UP supplementation not only promotes pathogen clearance but also shortens the duration of antibiotic therapy, thereby reducing antimicrobial resistance. It will also be critical to evaluate UP’s efficacy in vulnerable populations (i.e., immunocompromised patients and individuals with chronic inflammatory diseases) who may be particularly sensitive to antibiotic-induced mitochondrial dysfunction. Finally, we should emphasize the use of circulating biomarkers, such as GDF15, to identify patients requiring UP metabolic support to preserve immune competence during antibiotic treatment.

### Limitations of the study

This study comes with several limitations. First, the study cohort was relatively small and derived from a single clinical center, which may limit the generalizability of the results. Larger, multicenter cohorts will be necessary to further clarify the mechanisms and clinical relevance of these observations. This is particularly relevant for the flow cytometry analyses, which were performed in a small subset of patients. In addition, as surface marker expression does not necessarily reflect functional activity, intracellular cytokine staining will be important to directly link phenotype to effector function—specifically to determine whether CXCR3^+^ cells produce IFN-γ—thereby providing more direct evidence for a Th1-associated response. Second, patients received different classes of antibiotics with distinct mechanisms of mitochondrial toxicity, introducing potential heterogeneity in treatment effects. However, it has to be considered that the relatively modest sample size reflects the specific clinical context of this study. Patient inclusion required the detection of asymptomatic bacteriuria or positive sperm culture, conditions that are not systematically screened in routine clinical practice and are therefore identified only in a limited number of individuals. In addition, antibiotic selection was guided by national clinical guidelines for the management of these infections rather than being standardized within the study protocol, which resulted in the use of different antibiotic classes among participants.

Furthermore, although our data are consistent with a metabolic basis for the effects of UP supplementation, direct measurements of T cell metabolism were not performed, and it will be important to substantiate this interpretation. Another limitation of this study is that immune analyses were performed only at the end of treatment without paired baseline measurements. Consequently, we cannot exclude the possibility that some differences observed between groups reflect pre-existing immunological variability rather than treatment effects. However, patients were randomly allocated to treatment groups, reducing the likelihood of systematic baseline immunological differences. Notably, the convergence of independent immune readouts—including cytokine ratios, transcription factor expression, and flow cytometric phenotyping—provides internally consistent evidence of Th1 polarization, despite the absence of baseline immune measurements. Future studies incorporating paired baseline and post-treatment analyses will be required to confirm the treatment-related nature of the observed immune changes.

## Resource availability

### Lead contact

Requests for further information and resources should be directed to and will be fulfilled by the lead contact, Gaetano Villani (gaetano.villani@uniba.it).

### Materials availability

This study did not generate new unique reagents.

### Data and code availability

• Raw and processed data supporting the findings of this study have been deposited and are publicly available in Zenodo: https://doi.org/10.5281/zenodo.21217240.
• This paper does not report original code.
• Any additional information required to reanalyze the data reported in this paper is available from the lead contact upon request.

## Acknowledgments

We are grateful to the participants of our study.

## Author contributions

Conceptualization, P.S. and G.V.; data curation, M.R. and E.P.; formal analysis, S.D.S. and E.P.; funding acquisition, M.B., A. Moschetta, and G.V.; investigation, M.R., S.D.S., F.S., L.L., M.L.M., N.M., A. Mestice, and E.P.; methodology, M.R., S.D.S., and G.V.; project administration, M.R., E.P., and G.V.; resources, M.R., M.M., A. Vavallo, P.D., M.B., A. Moschetta, S.B., and G.V.; supervision, E.P. and G.V.; visualization, S.D.S. and E.P.; writing – original draft, S.D.S., E.P., G.V.

## Declaration of interests

G.V. and M.B. declare that they are inventors of a patent on the effects of UP on immune cells, and G.V. declares to hold an ownership interest in a company that possesses potential exploitation rights related to the above patent. P.S. declares that his wife, Martina Seibel, is the inventor of a patent on the ameliorative effects of UP during antibiotic therapies and that she is the CEO and holds an ownership interest in a company that possesses potential exploitation rights related to the above patent.

## STAR★Methods

### Key resources table

**Table:** 

REAGENT or RESOURCE	SOURCE	IDENTIFIER
**Antibodies**		

CD4 FITC	BD Biosciences	Cat# 345768; RRID: AB_2868797
CD196 (CCR6) PE, REAfinity™, Clone REA190	Miltenyi Biotec	Cat# 130-120-598; RRID: AB_2752157
CD194 (CCR4) REAfinity™, Clone REA279	Miltenyi Biotec	Cat# 130-120-456; RRID: AB_2784037
CD183 (CXCR3) APC, REAfinity™, Clone REA232	Miltenyi Biotec	Cat# 130-120-590; RRID: AB_2811347

**Biological samples**		

Serum and PBMCs	Urology, Andrology, and Kidney Transplantation Units of the University of Bar	N/A

**Chemicals, peptides, and recombinant proteins**		

Ficoll-Paque Premium	GE Healthcare Biosciences	GE17-5442-02
T Cell TransAct™, human	Miltenyi Biotec	130-111-160
7-AAD PerCP-Cy5.5	Becton, Dickinson and Company	559925

**Critical commercial assays**		

Human GDF15 ELISA assay	Thermo Fisher Scientific	#EHGDF15
Bio-Plex Pro Human Cytokines 27-plex Assay	Bio-Rad	#M500KCAF0Y
miRVana isolation kit	Thermo Fisher Scientific	AM1560
High-Capacity RNA-to-cDNA kit	Thermo Fisher Scientific	4387406

**Deposited data**		

Raw and processed data	Our laboratory	Zenodo: https://doi.org/10.5281/zenodo.21217240

**Oligonucleotides**		

Primers for qPCR, see Table S1	This paper	N/A

**Software and algorithms**		

GraphPad Prism v9.5	GraphPad Software	https://www.graphpad.com/
BioPlex Manager™ Software v6.2	Bio-Rad	https://www.bio-rad.com/
QuantStudio Design & Analysis	Thermo Fisher Scientific	https://www.thermofisher.com/
Diva software, v8.0.1	Becton, Dickinson and Company	https://www.bdbiosciences.com/

### Experimental model and study participant details

We conducted a pilot observational study enrolling 67 patients (Table 1) undergoing antibiotic therapy for asymptomatic bacteriuria or positive sperm culture according to EAU guidelines. As previously described,^17^ patients recruitment was performed in the Urology, Andrology, and Kidney Transplantation Units of the University of Bari between July 2017 and January 2023. The sample size was determined based on a preliminary power analysis, assuming a medium effect size, a significance level of 0.05, and a power of 80%. Patients who presented acute and/or life-threatening diseases (i.e., renal and hepatic failure), acute stress, infections, or were in treatment with medications affecting the number of leukocytes (i.e., corticosteroids) were excluded from the study. Other exclusion criteria were the following: allergy and/or adverse reactions to the prescribed drug or its excipient; pregnant and/or puerperal women; chronic systemic inflammatory diseases; immune-depression conditions; neoplastic diseases of recent onset (less than 10 years) and/or under chemotherapeutic treatment; infections sustained by Multi-Drug Resistant (MDR), Xtreme Drug-Resistant (XDR), Pan Drug-Resistant (PDR) microbes. Patients satisfying the eligibility criteria who interrupted the protocol were excluded from the study.

The enrolled patients underwent a complete blood count, urinalysis, and urine and/or semen culture (according to the clinical judgment) at baseline (T_0_), before starting any antibiotic treatment. Demographic and clinical data were collected. Then, participants were randomly allocated by an independent pharmacological consultant to either the “Antibiotic treatment plus Uridine and Pyruvate” (UP) group or the “Antibiotic treatment without UP” (control) group.

Patients in the UP group were instructed to take three doses of Uripyr® (each containing 150 mg of uridine and 2 g of pyruvate) per day, one hour before taking the antibiotic. Each subject was treated with a single antibiotic according to the culture test susceptibility results and the duration and dosage described by the best-accepted guidelines.

The mitotoxic antibiotics were administered at the following doses: levofloxacin 500 mg qd (7–10 days), ciprofloxacin 500 mg bid (7–10 days), tigecycline 50 mg bid (for 7 days), doxycycline 100 mg bid for the first day and then qd (7–10 days), linezolid 600 mg bid (10 days), erythromycin 1 g bid (10 days). Adverse effects, if any, were recorded. The use of any other phytotherapeutic agents for genital and urinary symptoms (e.g. Serenoa repens36) was also checked.

This study was conducted as an observational protocol of commercially available nutritional supplements (Italian Ministry of Health) and therefore did not require clinical trial registration. The Ethics Committee of the Azienda Ospedaliero-Universitaria Policlinico di Bari approved the study protocol (n. 5251). The study was conducted following the ethical principles of the Helsinki Declaration. Written informed consent for the use of clinical data was obtained from all participants in the study.

Experimenters were blind to group assignment and outcome assessment for all the experiments. All serum and PBMC samples were assigned anonymized identification codes before immunological analyses. Investigators were blinded to treatment allocation throughout all the analyses and data processing.

### Method details

#### Sample collection, serum and PBMCs isolation

At the end of the antibiotic treatment (T_END_), with or without UP supplementation, after overnight fasting, whole blood for serum and PBMCs isolation was collected by standardised venipuncture. Serum was obtained by centrifugation for 15 min at 1500x*g*, +4°C. PBMCs were isolated immediately after blood collection by density gradient centrifugation using Ficoll-Paque Premium (GE Healthcare Biosciences), and centrifuged for 30 min at 460x*g*, 18°C with no brake. Mononuclear cells were obtained from the central white band of the gradient, exhaustively washed in Phosphate-buffered saline (PBS). PBMCs were cryopreserved in 90% fetal bovine serum (FBS) supplemented with 10% dimethyl sulfoxide (DMSO) until further use.

The number of evaluable participants differed across downstream immunological assays, depending on the quantity and quality of biological material. No relevant demographic or clinical differences were observed between participants included in the immunological analyses and those not included.

#### PBMCs culture

After isolation, PBMCs were seeded at 1x10^6^/mL density and cultured in 100% autologous heat-inactivated (37°C for 30 minutes) serum. Then, cells were stimulated with 10μL T Cell TransAct® (Miltenyi Biotec, Germany) and kept in 5% CO_2_ at 37°C for 96 hours, and then subjected to different protocols.

#### ELISA analysis

ELISA quantification for Human GDF15 (cat. #EHGDF15, Thermo Fisher Scientific, USA) was performed on patients' sera following the manufacturer’s instructions.

#### BioPlex array

Cytokine concentrations in PBMC culture supernatants were measured using the BioPlex® platform (Bio-Rad, USA). Specifically, the Bio-Plex Pro Human Cytokines 27-plex Assay was employed to quantify the following analytes: FGF basic, Eotaxin, G-CSF, GM-CSF, IFN-γ, IL-1β, IL-1ra, IL-2, IL-4, IL-5, IL-6, IL-7, IL-8, IL-9, IL-10, IL-12 (p70), IL-13, IL-15, IL-17A, IP-10, MCP-1 (MCAF), MIP-1α, MIP-1β, PDGF-BB, RANTES, TNF, and VEGF, following the manufacturer’s protocol.

Briefly, samples were thawed on ice, centrifuged at 1,000 × g for 15 minutes, and diluted 1:4. Each 96-well plate was preloaded with 50 μL of fluorescently dyed magnetic microspheres covalently bound to specific antibodies. Subsequently, 50 μL of standards, controls, or diluted serum samples were added. Plates were incubated for 30 minutes at room temperature (RT) with shaking at 850 rpm, followed by three washes with wash buffer. Next, 25 μL of biotinylated detection antibody was added to each well, and the plates were incubated for another 30 minutes at RT under the same shaking conditions. After a further three washes, 50 μL of streptavidin–phycoerythrin conjugate was added, and plates were incubated for 10 minutes at RT with shaking, followed by a final set of three washes. Immunocomplexes were resuspended in 125 μL of assay buffer.

Fluorescence data were acquired using a BioPlex® 200 analyser (Bio-Rad, USA) and processed with BioPlex Manager™ Software version 6.2 (Build 175; Bio-Rad, USA). Median fluorescence intensity (MFI) values were used for quantitative analysis.

#### Gene expression

RNA extraction was performed on T_END_ PBMCs using the miRVana isolation kit (Thermo Fisher Scientific, USA) following the procedure for the extraction of total RNA, following the manufacturer’s instructions.

After assessing RNA integrity and purity, 500ng of RNA was retrotranscribed to cDNA using the High-Capacity RNA-to-cDNA kit (cat. 4387406, Thermo Fisher Scientific, USA) following the manufacturer’s instructions, and then eluted to 10ng/μl.

Gene expression analysis was performed on PBMC samples with sufficient RNA yield and quality. Real-time qPCR assays were performed in 96-well plates using the SsoAdvanced Universal SYBR Green Supermix (Bio-Rad, USA) via the QuantStudio5 machine (Thermo Fisher Scientific, USA), and the first analysis was performed using the QuantStudio Design & Analysis. Relative quantification was calculated via the ΔΔCT method, using GAPDH as a reference gene. All the primers used in this study are listed in Table S1.

#### Cytofluorimetric analysis

After 96h of culture, PBMCs were detached from the plate with cold D-PBS 1X (Gibco, New York, NY, USA) + 0.5 mM EDTA (Thermo Fisher Scientific, USA), washed with D-PBS 1X + 2mM EDTA (Thermo Fisher Scientific, USA) + 0.5%BSA (Sigma-Aldrich, USA), and stained for 20 min with CD4 FITC (cat. number 345768, BD Bioscences, USA), CD196 (CCR6) PE, REAfinity™, Clone REA190 (REF: 130-120-598, Miltenyi Biotec, Germany), CD194 (CCR4) REAfinity™, Clone REA279 (REF: 130-120-456, Miltenyi Biotec, Germany), CD183 (CXCR3) APC, REAfinity™, Clone REA232 (REF: 130-120-590, Miltenyi Biotec, Germany), according to the manufacturer’s instructions. Cell vitality was checked with 7-AAD PerCP-Cy5.5 (7-Aminoactinomycin D, Becton, Dickinson and Company, USA). Flow cytometer acquisition was performed using the FACSCanto II flow cytometer (Becton, Dickinson and Company, USA), and data were analysed using Diva software, version 8.0.1 (Becton, Dickinson and Company, USA). Gating strategy: forward scatter vs*.* side scatter for PBMCs gating. Single and live cells were selected from gated PBMCs and checked for positivity to CD4 (side scatter vs. CD4); CD4^+^ gated cells were analysed for CCR6 and CCR4 expression. CD4^+^CCR6^-^CCR4^-^ cells were checked for CXCR3 positivity. Flow cytometry was performed only on PBMC samples with adequate cell recovery and viability after thawing.

### Quantification and statistical analysis

Data are expressed as mean ± SEM. Statistical analysis was performed using GraphPad Prism software (v9.0, GraphPad Software Inc, CA). Outliers were detected by the ROUT method. Normality of data was assessed using the Shapiro-Wilk test. Comparison between 2 groups was performed by the Welch’s Test or the Mann-Whitney Test. Comparison between 4 groups was performed by 2-Way Anova, followed by Tukey’s test for multiple comparisons. A *p*-value <0.05 was considered significant. Significant effect was indicated by ∗ (∗*p* < 0.05, ∗∗*p* < 0.01). To account for multiple testing across the cytokine panel, *p*-values obtained from individual cytokine comparisons were adjusted for multiple comparisons using the two-stage Benjamini, Krieger, and Yekutieli false discovery rate (FDR) procedure (Q = 5%). Adjusted *p*-values (q-values) < 0.05 were considered statistically significant.

### Additional resources

The study protocol was approved by the Ethics Committee of the Azienda Ospedaliero-Universitaria Policlinico di Bari (n. 5251).
