# Supplementary material for: Uridine and pyruvate supplementation is associated with Th1 polarization during mitotoxic antibiotic therapy
Source: iScience. 2026 Aug 24;29(9):117230. doi: 10.1016/j.isci.2026.117230 (PMC13524768; doi:10.1016/j.isci.2026.117230)
Supplement: Supplementary file 1 [file mmc1.pdf]

iScience, Volume *29*

Supplemental information

Uridine and pyruvate supplementation

is associated with Th1 polarization

during mitotoxic antibiotic therapy

Stefania De Santis, Monica Rutigliano, Federica Simone, Letizia Larocca, Maria Laura Matrella, Martina Milella, Antonio Vavallo, Pasquale Ditonno, Nicola Marrano, Anna Mestice, Stefano Battaglia, Michele Battaglia, Antonio Moschetta, Peter Seibel, Elena Piccinin, and Gaetano Villani

SUPPLEMENTAL INFORMATION

Supplementary Table 1…………………………………………………………………………………… Page 2
Supplementary Table 2…………………………………………………………………………………… Page 3
Supplementary Figure 1………………………………………………………………………………… Page 4-5

Supplementary methods

Gene	Forward Primer (5'-3')	Reverse Primer (5'-3')
RORG	GAGGAAGTGACTGGCTACCAGA	GCACAATCTGGTCATTCTGGCAG
STAT6	CCTTGGAGAACAGCATTCCTGG	GCACTTCTCCTCTGTGACAGAC
TBX21	ATTGCCGTGACTGCCTACCAGA	GGAATTGACAGTTGGGTCCAGG

Supplementary Table 1. qPCR primer list.

Supplementary Results

Molecules	Abx	Abx+UP
Eotaxin	72.49±16.6	58.27±9
FGF	156.3±28.6	168.3±23.5
G-CSF	1937±415.2	2055±317.2
GM-CSF	32.62±11.63	88.31±27.18
IL-1β	63.19±26.7	59.11±19.6
IL-1ra	2241±583	9158±2266
IL-5	826.7±188.9	812.1±121.4
IL-9	1353±164.8	1249±111.5
IL-12	31.76±7.3	35.94±5.5
IL-15	672.5±94.2	693±76.8
IL-17	164.4±45.4	113.1±18.9
IP10	72.5±16.6	58.3±9.0
MCP-1	7277±1539	8629±1769
MIP-1a	2117±357.4	2502±358.7
MIP-1b	8585±2504	5081±1232
PDGF-bb	5653±671.8	4927±604.3
RANTES	19428±3401	12447±2116
VEGF	682.7±81.4	700.1±71.1

Supplementary Table 2. Cytokines, chemokines, and growth factors not significantly altered by Uridine and Pyruvate supplementation. PBMCs were isolated at the end of antibiotic treatment from patients treated with Abx alone vs. Abx+UP, stimulated to differentiate into T cells, and cultured in autologous serum for 96 hours. The concentration of these molecules was quantified by BioPlex analysis in PBMCs supernatants. Data reported as mean±SEM. No significant difference was observed in the level of these inflammatory molecules between the two groups. *Abx*: patients receiving antibiotic treatment alone, *Abx+UP*: patients receiving antibiotic therapy plus Uridine and Pyruvate supplementation

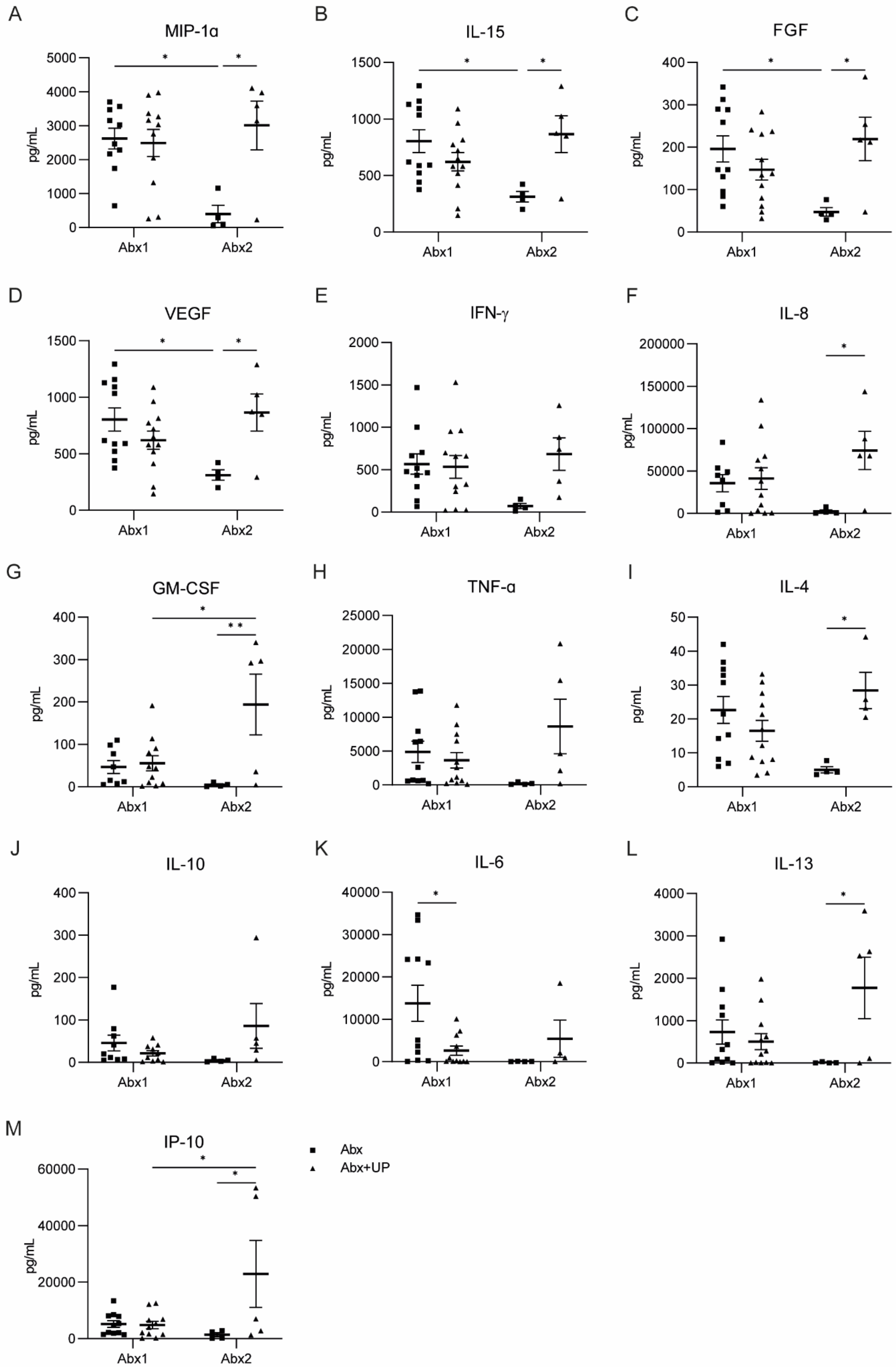
A
MIP-1α
B
IL-15
C
FGF
D
VEGF
E
IFN-γ
F
IL-8
G
GM-CSF
H
TNF-α
I
IL-4
J
IL-10
K
IL-6
L
IL-13
M
IP-10
pg/mL
Abx1
Abx2
Abx
Abx+UP

Supplementary Figure 1. Uridine and Pyruvate rescue cytokines, chemokines and growth factors levels in antibiotics that block mitochondrial protein synthesis, but not mitochondrial DNA gyrase. (A-I) Cytokine and growth factor concentrations in culture supernatants from PBMCs isolated at the end of antibiotic treatment from patients treated with Abx1 (antibiotics inhibiting the mitochondrial DNA gyrase) or Abx2 (antibiotics blocking mitochondrial protein synthesis) vs. Abx1/Abx2+UP. PBMCs were stimulated to differentiate into T cells, cultured in autologous serum for 96 hours, and cytokine production was quantified by BioPlex analysis. Data are shown as mean±SEM (*n=5-12/group*). Comparison between groups was performed by 2-Way Anova, followed by the Tukey's test for multiple comparisons (**p*>0.05). *Abx*: patients receiving antibiotic treatment alone, *Abx+UP*: patients receiving antibiotic therapy plus Uridine and Pyruvate supplementation.
